# Vitamin Concentrations in Human Milk Vary with Time within Feed, Circadian Rhythm, and Single-Dose Supplementation^1^,^2^,^3^,^4^

**DOI:** 10.3945/jn.116.242941

**Published:** 2017-02-15

**Authors:** Daniela Hampel, Setareh Shahab-Ferdows, M Munirul Islam, Janet M Peerson, Lindsay H Allen

**Affiliations:** 5 USDA–Agricultural Research Service Western Human Nutrition Research Center, Davis, CA;; 6 Department of Nutrition, University of California, Davis, Davis, CA; and; 7 Nutrition and Clinical Services Division, International Centre for Diarrhoeal Disease Research, Bangladesh, Mohakhali, Dhaka, Bangladesh

**Keywords:** lactation, human milk, sample collection, vitamins, circadian variation, acute supplementation effects

## Abstract

**Background:** Human milk is the subject of many studies, but procedures for representative sample collection have not been established. Our improved methods for milk micronutrient analysis now enable systematic study of factors that affect its concentrations.

**Objective:** We evaluated the effects of sample collection protocols, variations in circadian rhythms, subject variability, and acute maternal micronutrient supplementation on milk vitamin concentrations.

**Methods:** In the BMQ (Breast-Milk-Quality) study, we recruited 18 healthy women (aged 18–26 y) in Dhaka, Bangladesh, at 2–4 mo of lactation for a 3-d supplementation study. On day 1, no supplements were given; on days 2 and 3, participants consumed ∼1 time and 2 times, respectively, the US-Canadian Recommended Dietary Allowances for vitamins at breakfast (0800–0859). Milk was collected during every feeding from the same breast over 24 h. Milk expressed in the first 2 min (aliquot I) was collected separately from the remainder (aliquot II); a third aliquot (aliquot III) was saved by combining aliquots I and II. Thiamin, riboflavin, niacin, and vitamins B-6, B-12, A, and E and fat were measured in each sample.

**Results:** Significant but small differences (14–18%) between aliquots were found for all vitamins except for vitamins B-6 and B-12. Circadian variance was significant except for fat-adjusted vitamins A and E, with a higher contribution to total variance with supplementation. Between-subject variability accounted for most of the total variance. Afternoon and evening samples best reflected daily vitamin concentrations for all study days. Acute supplementation effects were found for thiamin, riboflavin, and vitamins B-6 and A at 2–4 h postdosing, with 0.1–6.17% passing into milk. Supplementation was reflected in fasting, 24-h postdose samples for riboflavin and vitamin B-6. Maximum amounts of dose-responding vitamins in 1 feeding ranged from 4.7% to 21.8% (day 2) and 8.2% to 35.0% (day 3) of Adequate Intake.

**Conclusions:** In the milk of Bangladeshi mothers, differences in vitamin concentrations between aliquots within feedings and by circadian variance were significant but small. Afternoon and evening collection provided the most-representative samples. Supplementation acutely affects some breast-milk micronutrient concentrations. This trial was registered at clinicaltrials.gov as NCT02756026.

## Introduction

The WHO recommends human milk as the sole food source for infants aged 0–6 mo (1). Therefore, an exclusively breastfed infant’s micronutrient status is dependent on the micronutrient concentration in breast milk, which can be greatly influenced by maternal dietary intake and status, especially in the case of vitamins (2, 3). However, very few data are available on nutrient concentrations in human milk in wealthier regions, and even less is known in poor populations. Although there is no reference range of concentrations for micronutrients in human milk, its micronutrient concentration has been estimated from published reports to use as the basis for making intake recommendations for breastfed infants and lactating women (4–7). However, the methods used for micronutrient analyses in human milk were rarely described sufficiently and were sometimes unsuitable for the complex human-milk matrix (8); in some cases, the results obtained with different methods were not comparable (3, 9, 10). We reported several validated methods for accurately analyzing multiple vitamins in human milk (11–13), which have now been used in various studies (14–21). Given the undeniable importance of adequate micronutrient supply for an infant’s growth and development, we still have only very limited knowledge about the extent to which micronutrient concentrations in breast milk are sufficient to meet an infant’s requirements.

Although correctly validated methods are necessary to adequately measure the micronutrient concentration in breast milk, the methods applied only reflect the concentration in the sample as provided. Procedures for collecting milk samples vary greatly among studies (e.g., opportunistic sample collection, samples collected after a period of no-breastfeeding or at some time during a feeding), samples may be with or without maternal supplementation, and they often are not accompanied by maternal dietary status data (15, 16, 19, 22–32). To what extent these variations influence the micronutrient concentration of milk has not been assessed. Thus, a standardized and optimized milk collection method is essential for a true assessment of breast-milk micronutrients.

In this study, we collected milk from 18 Bangladeshi mothers during every feeding from the same breast over 24 h, and continued the sample collection protocol for another 48 h during which the mothers received a single dose of a micronutrient supplement with breakfast (0800–0900). Samples were analyzed for retinol (vitamin A), tocopherol (vitamin E), thiamin (vitamin B-1), riboflavin (vitamin B-2), nicotinamide (vitamin B-3), vitamin B-6, and vitamin B-12 to examine *1*) within-feeding variation, *2*) circadian variation and contributions due to subject variability, *3*) acute short-term effects of maternal supplementation on milk vitamin concentrations, and *4*) the optimal time for collection to determine which milk samples best represented the daily median vitamin concentrations in milk.

## Methods

### 

#### Subjects and sample collection.

**FIGURE 1:**
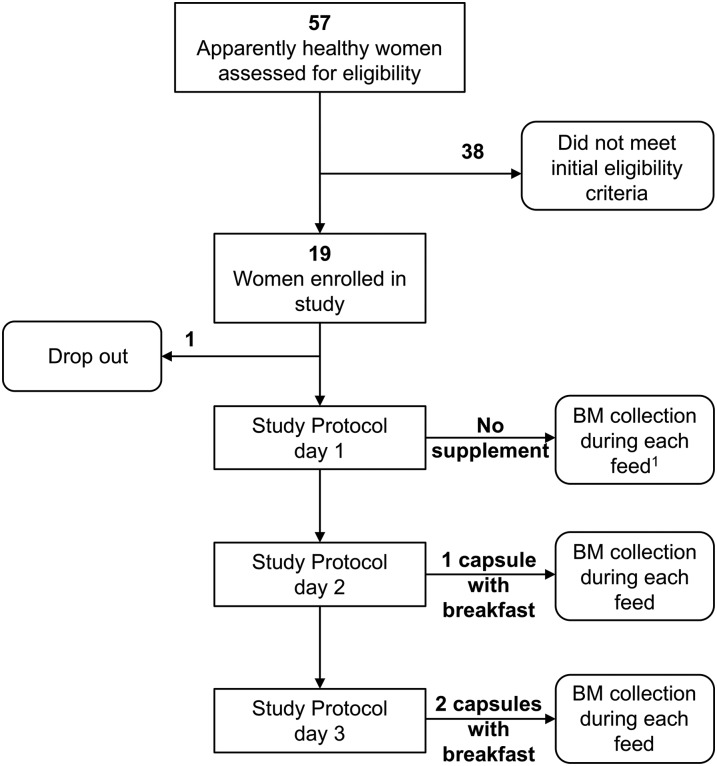
Flowchart of study participants in the Breast-Milk-Quality Study. Eighteen apparently healthy Bangladeshi women were enrolled in the study. ^1^Three aliquots of breast milk were collected from the same breast during every feeding for the duration of the study (I: the first 2 min into the feeding; II: the remainder of the feeding; III: full-breast aliquot obtained by combining I and II). BM, breast milk.

For this 3-d observational supplementation trial, 18 lactating mothers of infants aged 2–4 mo were recruited at the International Centre for Diarrhoeal Disease Research, Bangladesh (ICDDR,B)^8^, in Dhaka between October 2013 and February 2014. The recruitment methods and flow of participants are shown in **Figure 1**. This research was approved by the Institutional Review Board of the University of California, Davis, and ICDDR,B internal review boards; and consent was obtained by the Bangladesh principal investigator (clinicaltrials.gov; NCT02756026). The women provided informed written consent in Bangla for themselves and their infants. The number of participants was limited by the availability of resources. To be eligible for the study, the women had to have a BMI (in kg/m^2^) >18.5 and be at 2–4 mo of lactation and exclusively breastfeeding ≥12 times/d. Furthermore, women and their infants had to have no pre-existing health problems and if within the last week there had to be no signs of cold and cough, difficulty breathing, fever, diarrhea, and, in the mother, no mastitis.

The women and their infants arrived at the Clinical Trial Unit of the ICDDR,B during the day before the first study day and stayed in the unit for the duration of the study. Meals consisting of rice and *dahl* (a local lentil dish) were low in micronutrients and provided throughout the study. There were no differences between the meals for each day. The first breast-milk sample was collected from the overnight-fasted mother in the morning of the first study day between 0800 and 0859 by the study nurses. During every feeding, milk was collected by using the Medela Symphony electronic hospital-grade breast pump (Medela) from the same breast for 24 h, allowing the infant to suckle from the other breast. Milk expressed during the first 2 min (aliquot I) was collected separately by a specially designed funnel attached to the nipple shield into a 50 mL conical tube (Corning, Inc.); the remainder (aliquot II) of the breast content was collected into the pump’s milk bottle provided by the manufacturer until the breast was emptied. A third aliquot (aliquot III) was saved by transferring the remaining milk from collection (I) into the bottle containing aliquot II. From each aliquot (I, II, and III), 2 × 1 mL (sample and backup) were gently mixed before transferring into 2-mL amber screw-cap tubes (Fisher Scientific) by using plastic disposable transfer pipettes (Fisher Scientific) and immediately stored at −20°C on site. After aliquots were taken, the remaining milk was available for spoon-feeding by the mother. The same milk-collection protocol was followed on days 2 and 3, but women received supplements containing ∼1 time and ∼2 times, respectively, the US-Canadian RDA in a capsule (Nutri-Fem; Thorne Research; **Supplemental Table 1**) provided during breakfast by the study personnel. On day 4, a final fasting breast-milk sample was obtained before the participants were discharged from the Clinical Trial Unit. Milk volume was not recorded. The milk samples (aliquots I, II, and III) were shipped on dry ice to the USDA–Agricultural Research Service Western Human Nutrition Research Center in Davis, California, for analysis, and the backup samples remained on site at the ICDDR,B.

#### Biochemical analyses.

Unless otherwise stated, all milk aliquots (I, II, and III) were analyzed by using the following methods. Riboflavin, FAD, nicotinamide, and pyridoxal were analyzed as previously described (13), with a few modifications. Samples were analyzed after protein precipitation and removal of nonpolar constituents with methyl-tert-butyl ester (Fisher Scientific) with the use of a Waters Alliance 2695 HPLC coupled to a Micromass Micro Quattro mass spectrometer (Waters). Analytes were separated by using a Waters Atlantis T3 (3 μm, 2.1 mm × 75 mm) column guarded by a Thermo Scientific BDS-hypersil C18 Javelin guard (3 μm, 3 mm × 20 mm) (Thermo Fisher Scientific) maintained at 40°C.

Free thiamin, thiamin monophosphate, and thiamin pyrophosphate were analyzed via HPLC–fluorescence detector using an Agilent 1200 Series HPLC system (Agilent) after precolumn derivatization to their thiochrome esters (11). Retinol, β-carotene, and α-tocopherol were analyzed as described (33), with some modifications: only 100-μL samples were used for analysis with an Agilent Eclipse Plus C18 column (3.5 μm, 2.1 mm × 100 mm) guarded by a Thermo Scientific BDS-hypersil C18 Javelin guard (3 μm, 3 mm × 20 mm) maintained at 30°C. Twenty-five microliters were injected at a flow rate of 0.6 mL/min and a total run time of 4.5 min. Vitamin B-12 in breast milk was analyzed as previously described by using the Siemens Immulite automated, quantitative immuno-analyzer (12).

Creamatocrit and fat contents were determined by using Creamatocrit Plus (Medela) according to the manufacturer’s protocol. A control strip provided by the manufacturer was used to verify the tube reader’s accuracy.

#### Statistical analyses.

The outcomes were concentrations of thiamin (vitamin B-1; free thiamin + thiamin monophosphate + thiamin pyrophosphate), riboflavin (vitamin B-2; riboflavin + FAD), niacin (vitamin B-3; nicotinamide), vitamin B-6 (pyridoxal), vitamin B-12, vitamin A (retinol + β-carotene), and vitamin E (α-tocopherol), and fat-adjusted vitamins A (vitamin A^fat^; retinol + β-carotene per gram of milk fat) and E (vitamin E^fat^; α-tocopherol, the active form of vitamin E, per gram of milk fat) in breast milk. SAS for Windows, release 9.4 (SAS Institute), was used for all statistical analyses. Logarithmic transformations were performed on riboflavin, niacin, vitamin B-6, vitamin A, vitamin A^fat^, vitamin E^fat^, fat, and the square root transformation vitamin E to normalize the skewed data. No transformation was needed for thiamin and vitamin B-12. All of the aliquots (I, II, and III) obtained on day 1 were used to examine the differences in concentrations between these aliquots (within-feeding variation), because no supplements were consumed, with the use of general linear models (GLM procedure; SAS), with time, aliquot, and subject included as fixed categorical effects. This approach showed that aliquot III is the preferred aliquot to be collected; thus, all subsequent statistical analyses were carried out with the use of aliquot III only.

The GLM procedure was also used to explore the differences between the fasted samples of each day (days 1–4; acute supplementation effects on fasted samples), with day and subject used as fixed categorical effects. Mixed-model analysis (MIXED procedure; SAS) was used to explore the differences in concentrations by day (days 1–3; acute short-term effects of supplementation) to identify the timing of the sample whose concentration was closest to the daily overall concentrations (optimal time for collection) and to estimate the percentage of variance due to hourly fluctuation and due to within- and between-subject variability. All mixed models included subject as a categorical random effect. Analyses involving multiple comparisons were adjusted with Tukey-Kramer test. To compare vitamin concentrations of a given time interval with the overall daily concentrations, the samples (aliquot III) from all subjects combined for a given day were categorized on the basis of time of collection (1-h time intervals during 0800–2000 and 2-h time intervals during 2000–0800). *P* values <0.05 were considered to be significant. Median concentrations for each time interval were used for solely illustrative purposes in the tables and figures.

#### Efficacy of maternal supplementation.

The efficacy of maternal supplementation was estimated for vitamins in breast milk that showed significant acute short-term supplementation effects (*P* < 0.05). Therefore, the increases in vitamin concentrations due to supplementation were estimated by using the differences in daily median concentrations for days 1–3 (day 2 compared with day 1, day 3 compared with day 1, day 3 compared with day 2). After adjusting to the average milk intake for infants of 0.78 L/d, this median daily increase in concentration was compared with *1*) the respective amount provided by the supplement and *2*) the respective values used to set the Adequate Intake. To examine the maximum dose provided to the infant in a single feeding, the highest median concentrations of a responding vitamin at any given time interval within a day were used to estimate the maximum amount provided within a feeding. Calculations were based on an average feeding frequency in this study of 10 feedings/d and 780 mL milk consumed by the infant/d.

## Results

### 

#### Maternal characteristics at initial visit.

The women recruited for the study were housewives between 18–26 y of age with a BMI in the normal range (**Table 1**). Of the participants, 83.3% had some level of education and 94.4% lived in a household with an income above the minimum wage. Only 1 participant reported current vitamin use, whereas 83.3% of the women reported the use of various supplements during pregnancy.

**TABLE 1:** Characteristics of Bangladeshi mothers 2–4 mo postpartum participating in the BMQ study^1^

Characteristics	Values
Maternal characteristics	
Age, y	20 (18, 22)
Weight, kg	47 (42, 53)
Height, cm	149 (145, 152)
BMI, kg/m^2^	22 (19, 24)
Any education, %	83.3
Income > minimum wage, %	94.4
Current vitamin use, %	5.6
Supplements in pregnancy, *n* (%)	
Iron + calcium + VD	7 (38.9)
Iron	3 (16.7)
Iron + BV	1 (5.6)
Iron + MNs	1 (5.6)
Iron + calcium	1 (5.6)
Ca + VD	1 (5.6)
MNs	1 (5.6)
No supplement use, *n* (%)	3 (16.7)

1 Values are medians (IQRs) unless otherwise indicated; *n* = 18. BMQ, Breast-Milk-Quality; BV, B-vitamin complex; MN, micronutrient; VD, vitamin D.

#### Differences between aliquots I, II, and III within a day.

**FIGURE 2:**
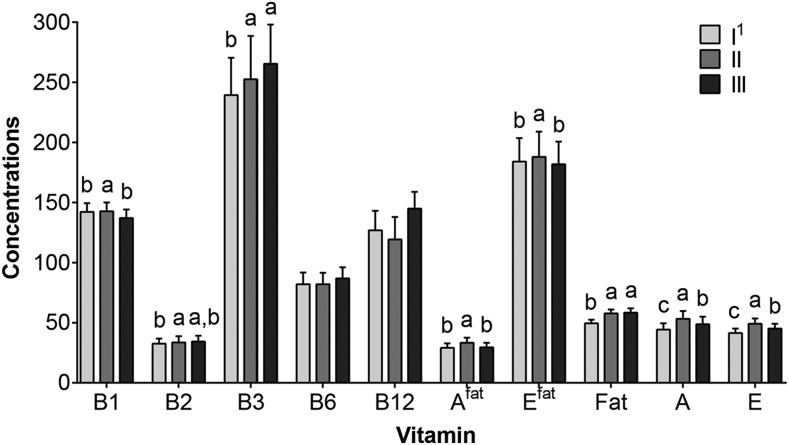
Differences between milk aliquots collected within a feeding during a day without maternal supplementation. Values are medians (95% CIs); *n* = 187 from 18 apparently healthy Bangladeshi mothers at 2–4 mo of lactation. Labeled medians without a common letter differ, *P* < 0.05. I^1^, first 2 min of the feeding; II, remainder of the feeding; III, full-breast aliquot obtained by combining I and II. General linear models were used to explore the differences in concentration between aliquots on day 1. All concentrations are μg/L except for B12 (ng/L), A^fat^ and E^fat^ (nmol/L), Fat (g/L), A (μg/100 mL), and E (μg/10 mL). A, vitamin A; A^fat^, vitamin A adjusted for milk fat; B1, thiamin; B2, riboflavin; B3, niacin (nicotinamide); B6, vitamin B-6; B12, vitamin B-12; E, vitamin E; E^fat^, vitamin E adjusted for milk fat; Fat, milk fat.

The differences in vitamin concentrations between the collected aliquots I, II, and III were examined on day 1, when no supplementation was provided. No significant differences were found for vitamins B-6 and B-12 **(****Figure 2**). When compared with the full-breast aliquot III, aliquot I was significantly lower in concentrations of niacin, fat, vitamin A^fat^, and vitamin E^fat^, whereas aliquot II was significantly higher for thiamin and vitamins A, E, A^fat^, and E^fat^. None of these differences were large (>20% between aliquots). Thus, any further analyses included only the concentrations obtained from aliquot III.

#### Circadian variation and subject variability.

Regardless of the day, the circadian variation in concentration was significant for all vitamins (*P* < 0.001), except for vitamins A and E when adjusted for milk fat. However, the contribution of the circadian variation to the total variance was <10% for all vitamins, but increased considerably with maternal supplementation for riboflavin (36%) and vitamin B-6 (23%) (**Table 2**, **Supplemental Table 2**).

**TABLE 2:** Circadian variations in breast-milk concentrations for thiamin, riboflavin, niacin, fat, and vitamins B-6, B-12, A, and E for days 1–3^1^

		Vitamin or fat, %									
Time (24 h)^2^	*n*	Thiamin	Riboflavin	Niacin	Vitamin B-6	Vitamin B-12	Vitamin A	Vitamin E	Fat	Vitamin A^fat^	Vitamin E^fat^
Day 1											
0800–0859	17	85*	70*	82	94	121	80	97	90	101	103
0900–0959	3	95	39	42	126	34	119	98	117	101	91
1000–1059	12	91	133	102	103	74*	138	119	105	94	105
1100–1159	10	101	83	122	106	146*	98	101	96	81	116
1200–1259	6	118*	169*	75	116	61	84	107	87	108	116
1300–1359	11	100	96	79	94	117	107	112	114	108	83
1400–1459	9	103	95	166	131	137*	117*	142*	123*	96	101
1500–1559	8	115	101	73	106	119	120	105	133	82	89
1600–1659	12	85	121	112	88	94	119	101	93	132	108
1700–1759	9	108	102	99	107	75	71	87	108	82	93
1800–1859	9	81	86	117	92*	162	146	136	112	107	111
1900–1959	7	107	153	125	136*	146	90	105	96	185	109
2000–2159	20	103	111	88	105	126	121	96	111	127	82
2200–2359	12	124	131	93	113	105	126	82	89	122	90
0000–0159	12	98	79	112	69*	75	69*	83*	78*	80	93
0200–0359	11	96*	79*	50*	67*	59*	69	82	80	81	90
0400–0559	10	108*	146	102	67	63*	109*	81	80	128	95
0600–0759	9	76*	61*	80*	71*	108	87	84	90	92	108
Daily median^3^	187	137	34.3	265	86.9	145	48.8	45.1	58.4	29.4	182
Variance due to circadian fluctuation, %		3.7	2.1	3.0	3.0	8.6	5.4	5.5	8.7	<0.5	<0.5
Variance due to between-subject variability, %		81.8	86.3	73.7	76.8	54.6	56.7	48.0	18.7	67.2	58.4
Variance due to within-subject variability, %		14.5	11.6	23.3	20.2	36.8	37.9	46.5	72.6	32.8	41.6
Day 2											
0800–0859	17	90*	69*	69	76	92	126	118*	96	120	109
0900–0959	7	100	159*	116	105*	55	98	123	111	88	90
1000–1059	8	86	127*	85	86	145	132	130	120*	118	104
1100–1159	10	107*	167*	97	127*	54	119	113	100	112	118
1200–1259	12	97*	136*	103*	119*	131	99*	125*	111	87	114
1300–1359	4	117	220	155	128	172*	210	93	106	203	68
1400–1459	8	110	106	100	78	132	120*	113*	131*	91	81
1500–1559	10	112	113	115	128	85	146	103	104	129	98
1600–1659	10	88	80	90*	108	156	113*	147*	100	92	110
1700–1759	10	110	95	115	93	118	119	99	104	134	78
1800–1859	4	105	119	154	82	89	107	117	129	82	80
1900–1959	8	76	68	67	91	123	101	116	79	155	112
2000–2159	16	111	97	101	85	72	92	94	96	92	86
2200–2359	17	93	100	109	103	100	101	79	103	102	119
0000–0159	10	94*	78	80	59*	84	76*	77*	64*	110	127
0200–0359	10	82*	62*	66*	111	89	69*	87*	76*	84	113
0400–0559	5	113	125	108*	113	72	111	70	74	155	84
0600–0759	13	74*	76*	75*	114	74	78*	91	83	91*	85
Daily median^3^	179	160	45.1	229	126	146	53.2	42.3	55.7	32.3	190
Variance due to circadian fluctuation, %		3.4	6.6	3.1	5.5	3.2	6.8	6.9	15.4	<0.5	<0.5
Variance due to between-subject variability, %		85.3	79.6	79.1	74.5	49.9	66.1	51.8	8.4	67.7	46.9
Variance due to within-subject variability, %		11.3	13.8	17.8	19.9	46.9	27.2	41.2	76.2	32.3	53.1
Day 3											
0800–0859	16	86*	61*	96	69*	88	91	107	106	85	88
0900–0959	7	99	167*	97	83	61	107	135	119	100	113
1000–1059	8	101	972*	105	228*	102	96	107	129	108	86
1100–1159	10	107	476*	88	134*	82	76	90	89	93	111
1200–1259	8	99*	188*	168*	210*	125*	139*	131*	101	142*	102
1300–1359	11	132*	288*	78*	283*	84	114	118	106	115*	93
1400–1459	6	95	114	117	183*	86	142*	100*	77	153*	137*
1500–1559	14	118*	133	140*	107	118*	125*	111	117*	101	107
1600–1659	6	83	70	81*	111	124	114	120	104	72	108
1700–1759	7	116	151	103	102*	131	190	89	101	188	97
1800–1859	9	107	94	162	126	132	88	101	112	93	97
1900–1959	6	121	121	123	117	101	123	71	93	97	76
2000–2159	16	94	71*	87	82	83	88	108	98	100	98
2200–2359	17	89	62*	126	80*	113	88	97	97	89	105
0000–0159	4	97*	57*	66	54*	61	78	86	85	80	95
0200–0359	9	98*	86	108*	77*	61*	75	80	97	104	83
0400–0559	10	83*	42*	85*	64*	98	64	87	88	100	116
0600–0759	9	80*	41*	92*	94*	126*	71*	83	70*	72	126*
Daily median^3^	173	166	64.7	193	214	145	60.2	41.9	56.0	37.9	172
Variance due to circadian fluctuation, %		8.8	40.5	6.2	23.9	3.9	5.8	2.0	7.0	<0.5	0.9
Variance due to between-subject variability, %		70.0	30.5	82.4	59.3	66.9	59.5	48.1	23.9	64.3	59.9
Variance due to within-subject variability, %		21.2	29.0	11.4	16.8	29.2	34.8	49.9	69.1	35.4	39.2

1 Values are relative medians (%) compared with the daily median unless otherwise indicated. Relative median: for each day, medians were determined for each time interval as shown and divided by the overall daily median. Values were obtained from mean values of aliquot III (full-breast aliquot) of the milk sample collection from 18 healthy Bangladeshi mothers at 2–4 mo of lactation. Mixed-model analysis of transformed values was used to identify the most-representative sample collection time and to estimate the percentage of variance due to hourly fluctuation and subject variability. *Different from the daily median, *P* < 0.05. Fat, milk fat; Vitamin A^fat^, vitamin A adjusted for milk fat; vitamin E^fat^, vitamin E adjusted for milk fat.

2 The optimal times for collection with no significant differences of concentrations from the daily median are as follows: Day 1: 0900–0959, 1300–1359, 1500–1759, 2000–2359; Day 2: 1500–1559, 1700–2359; Day 3: 1800–1959.

3 Vitamin A (μg/100 mL); vitamin A^fat^ (nmol/g milk fat); thiamin, riboflavin, niacin, and vitamin B-6 (μg/L); vitamin B-12 (ng/L); vitamin E (μg/10 mL); vitamin E^fat^ (nmol/g milk fat); fat (g/L).

On day 1 (no supplements), vitamin E^fat^ showed the lowest range of variation throughout a day (82–116%), whereas the greatest range was observed for riboflavin (39–169%). The optimal collection times were 0900–0959, 1300–1359, 1500–1759, and during the evening, 2000–2359 (Table 2).

On day 2, the first day of supplementation (1 capsule), thiamin varied least from the daily median (74–117%), whereas riboflavin showed the greatest range (62–220%). Increases in concentrations of >200% of the daily median were observed for riboflavin within 4 h after the supplement was taken. The optimal collection times were between 1500–1559 and 1700–2359 showing no significant differences between the measured vitamin concentrations during these time intervals and the overall daily median concentrations.

The second day of using a higher dose of supplement (day 3) showed the same pattern for the vitamins with the smallest range (thiamin: 80–132%) and the greatest range (riboflavin: 41–972%). Riboflavin and vitamin and B-6 increased by >200% of their daily median concentrations within 2–4 h after supplementation. Differences in concentrations for all vitamins from their daily concentrations were not significant between 1800 and 1959.

Although circadian variation represented only a minor contribution to the total variance, the between-subject variability accounted for a majority of the total variance for most of the vitamins on all days (46.9–86.3%; Table 2), with the exception of riboflavin on day 3 (30.1%) when the variance of the circadian variation increased to 36%, most likely due to maternal supplementation. The between-subject variability was consistently lower for fat than for the vitamins on all days (8.9–23.8%), whereas the within-subject variability was lower for vitamins (11.3–53.1%; Table 2) than for the fat content (69.1–76.2%).

#### Acute effects of supplementation.

**FIGURE 3:**
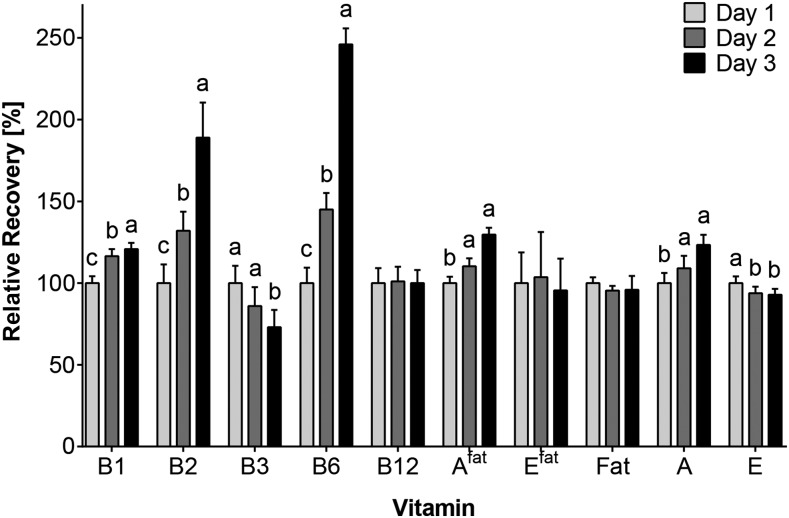
Relative daily concentrations of vitamins and fat in breast milk during maternal supplement consumption on days 2 (1 capsule) and 3 (2 capsules). Values are medians (95% CIs); day 1, *n* = 187; day 2, *n* = 179; day 3, *n* = 173 from 18 women. Labeled medians without a common letter differ, *P* < 0.05. Relative median concentrations (%) are based on median concentrations on day 1 (set to 100% by default) and median concentrations on days 2 and 3. Only the full-breast aliquots (aliquot III) were considered. The mixed-model analysis was used to examine for differences in concentrations by day. A, vitamin A; A^fat^, vitamin A adjusted for milk fat; B1, thiamin; B2, riboflavin; B3, niacin (nicotinamide); B6, vitamin B-6; B12, vitamin B-12; E, vitamin E; E^fat^, vitamin E adjusted for milk fat; Fat, milk fat.

Thiamin, riboflavin, and vitamins B-6, A, and A^fat^ concentrations in breast milk were significantly increased when supplements were consumed (**Figure 3**), with median increases >180% (riboflavin and vitamin B-6) and 120–130% (thiamin and vitamins A and A^fat^) compared with the median baseline value of day 1. Niacin and vitamin E both showed significantly lower concentrations on days when supplements were consumed. No effects of acute supplementation were found for vitamin B-12 and E^fat^ concentrations.

Even though the increase in all 4 vitamins was significant and substantial from both doses, only 0.05–0.35% of thiamin, 0.54–0.6% of riboflavin, 1.0–1.14% of vitamin B-6, and 4.85–6.17% of vitamin A in the supplements were secreted into the breast milk and subsequently available to the infant (**Table 3**). All of the vitamins except for thiamin showed a positive linear dose-response relation; thiamin secretion into the milk was 4 times more efficient on day 1 compared with day 2. However, when compared with their Adequate Intakes, only 2.3–11.1% of thiamin, 2.8–7.9% of riboflavin, and 8.6–22.3% of vitamin A were transferred to the milk via maternal supplement, whereas 30.7–98.9% of the Adequate Intake was provided by maternal vitamin B-6 supplementation. The maximum dose provided in 1 single feeding ranged between 1.5% and 13.9% of the Adequate Intake when no supplements were provided, which increased to 8.2–35% when supplements were given.

**TABLE 3:** Estimate of the acute effects of supplementation on thiamin, riboflavin, and vitamins B-6 and A in fasting milk samples collected from healthy Bangladeshi women who consumed 1 (day 2) and 2 (day 3) capsules of the micronutrient supplement^1^

	Thiamin	Riboflavin	Vitamin B-6	Vitamin A
Day, μg/L				
1	137 (108, 179)^2^	34.3 (17, 56)	86.9 (54, 117)	532 (353, 829)
2	160 (113, 185)	45.1 (26, 80)	126 (80, 191)	566 (409, 897)
3	166 (131, 199)	64.7 (33, 113)	214 (146, 281)	651 (466, 930)
Increase, μg/L				
On day 2	22.6	10.8	39.3	34.0
On day 3 vs. day 2	6.0	19.6	87.5	84.7
On day 3 vs. day 1	28.6	30.4	127	119
Increase based on infant intake (0.78 L/d), μg/d				
On day 2	17.7	8.4	30.7	26.5
On day 3 vs. day 2	4.6	15.3	68.3	66.1
On day 3 vs. day 1	22.3	23.7	98.9	92.6
Supplemental amount received, mg				
Day 2	5	1.4	3	0.56
Day 3 vs. day 2	10	2.8	6	1.1
Day 3 vs. day 1	15	4.2	9	1.7
Supplement secreted into breast milk,^3^ %				
Day 2	0.35	0.60	1.0	4.7
Day 3 vs. day 2	0.05	0.54	1.1	6.0
Day 3 vs. day 1	0.14	0.56	1.1	5.5
Supplement amount received,^4^ % of Adequate Intake				
Adequate Intake for infants aged 0–6 mo, μg/d	200	300	100	400
Day 2, % of Adequate Intake	8.8	2.8	30.7	6.6
Day 3 vs. day 2, %	2.3	5.1	68.3	16.6
Day 3 vs. day 1, %	11.1	7.9	98.9	23.2
Median maximum concentration,^5^ μg/L				
Day 1	169	58.1	118	711
Day 2	187	99.1	162	1117
Day 3	211	438	449	1142
Amounts provided at maximum,^6^ μg/feeding (% of Adequate Intake)				
Day 1	13.2 (6.6)	4.5 (1.5)	9.2 (9.2)	55.4 (13.9)
Day 2	14.6 (7.3)	7.7 (4.7)	12.6 (12.6)	87.1 (21.8)
Day 3	16.4 (8.2)	34.2 (11.4)	35.0 (35.0)	89.1 (22.3)

1 *n* = 18. Samples were obtained from milk aliquot III collected from healthy Bangladeshi mothers at 2–4 mo of lactation.

2 Median; IQR in parentheses (all such values).

3 Increase based on infant intake compared with the supplemental amount received.

4 Increase based on infant intake compared with Adequate Intake values (4, 6).

5 Highest median concentration measured at any given time interval within a day.

6 Estimated amount of vitamin in a single feeding at maximum median concentrations based on an average of 10 feedings/d and 780 mL daily consumption = 78 mL, presented as micrograms per feeding (% of Adequate Intake).

#### Supplementation effects in fasting milk samples.

Nutrient concentrations in the fasting milk samples collected on each study day (days 1–4) were compared to evaluate possible supplementation effects on fasting milk samples, which also represented samples collected at the maximum time after the supplements were consumed. No significant differences were observed for thiamin and vitamins B-12, A^fat^, E^fat^, and A (**Table 4**). Vitamin B-6 concentrations increased linearly after supplements were consumed, whereas milk concentrations of riboflavin increased on day 3 and 4 compared with day 1. Concentrations on day 2 did not differ from day 1 or days 3 and 4. Lower concentrations were found for niacin and vitamin E, but only on day 4.

**TABLE 4:** Concentrations of all nutrients analyzed in fasting milk samples collected from healthy Bangladeshi women who consumed 1 (day 2) and 2 (day 3) capsules of the micronutrient supplement^1^

Nutrient	Day 1 (*n* = 17)	Day 2 (*n* = 17)	Day 3 (*n* = 16)	Day 4 (*n* = 18)	*P*
Thiamin, μg/L	116 (102, 156)	144 (109, 176)	143 (106, 195)	152 (117, 174)	0.07
Riboflavin, μg/L	24^b^ (15, 41)	31^a,b^ (14, 55)	40^a^ (21, 70)	35^a^ (21, 49)	0.06
Niacin, μg/L	219^a,b^ (161, 295)	158^b^ (138, 434)	185^a,b^ (114, 338)	159^a^ (87, 286)	0.03
Vitamin B-6, μg/L	81^c^ (67, 103)	96^b,c^ (65, 154)	148^a,b^ (109, 246)	211^a^ (164, 283)	<0.001
Vitamin B-12, ng/L	175 (85, 224)	134 (75, 199)	128 (78, 198)	103 (78, 180)	0.26
Vitamin A^fat^, μmol/g fat	30 (18, 54)	39 (26, 56)	33 (22, 52)	39 (25, 56)	0.09
Vitamin E^fat^, μmol/g fat	188 (119, 237)	207 (183, 256)	151 (126, 213)	196 (153, 284)	0.36
Fat, g/L	52^a,b^ (37, 70)	54^a^ (48, 68)	60^a^ (49, 67)	39^b^ (30, 52)	0.03
Vitamin A, μg/L	391 (314, 561)	669 (371, 907)	547 (447, 762)	391 (314, 561)	0.11
Vitamin E, μg/L	4.4^a,b^ (3.5, 6.1)	5.0^a^ (3.9, 7.4)	4.5^a,b^ (3.3, 5.8)	3.2^b^ (2.1, 3.8)	0.06

1 Values are medians (IQRs); *n* = 18. Samples were obtained from milk aliquot III collected from healthy Bangladeshi mothers at 2–4 mo of lactation. Samples were collected between 0800 and 0859 before breakfast, ∼24 h after the last supplement consumption, and before the dose of the day. *P* values were obtained by using a generalized linear model. Labeled medians in a row without a common superscript letter differ, *P* < 0.05. Fat, milk fat; vitamin A^fat^, vitamin A adjusted for fat; vitamin E^fat^, vitamin E adjusted for fat.

## Discussion

Although numerous studies that required breast-milk collection have been conducted over decades, little systematic attention was given to the influence of the collection conditions on breast-milk nutrient concentrations. Moreover, the differences in sample collection methods exacerbated uncertainties in the comparison of the results between different studies.

The objective of the BMQ (Breast-Milk-Quality) study was to determine which milk aliquot collected best represented the daily median milk vitamin concentration and how single-dose maternal supplement consumption affects milk vitamin concentrations. Thus, the study was not designed to detect differences in milk composition on the basis of stage of lactation, to estimate infant intakes, or to evaluate long-term supplementation effects. Because the breast milk was collected on the basis of the infant’s desire to breastfeed, the time points of milk collection were irregular and produced a different number of samples for the different time intervals and therefore resulted in variable power to detect differences. Because the milk volume was not recorded, the actual infant intake of the micronutrients could not be estimated.

We found significant differences between the 3 aliquots (I, II, and III) collected within a feeding for some of the vitamins analyzed (thiamin, riboflavin, niacin, vitamin A, vitamin A^fat^, vitamin E^fat^, and fat), whereas others (vitamins B-6 and B-12) were not affected. Although Greibe et al. (29) found higher amounts of vitamin B-12 in hindmilk than in foremilk, Trugo and Sardinha (34) found no differences in vitamin B-12 concentration within feedings, between breasts, or over the day. The latter report is in agreement with our findings. Hence, depending on the nutrient of interest and the aliquot collected, concentrations obtained for some vitamins may under- or overestimate the actual concentrations in milk by ≤20%, indicating that an aliquot from the full-breast volume is the preferred milk sample. Circadian variation was significantly influenced by time of sample collection within a day, but the contribution to the total variance was relatively small. In contrast, the variation due to between-subject variability represented the main contributor to the total variance. Other factors, such as recent or current intake and/or vitamin status and genetics, are likely to influence the total variances in vitamin concentrations among women, which need to be quantified in future studies.

When supplements were consumed, the contribution of circadian variance to the total variance increased, implicating an acute effect of maternal supplementation at least for some of the vitamins analyzed. However, with or without supplements, the best representative time for sample collection was during the late afternoon and evening hours. Without supplementation, the morning hours (0900–0959) and early afternoon hours (1500–1559) were also acceptable for sample collection based on their lower variation in vitamin concentrations, whereas increases in the concentration of riboflavin and vitamin B-6 in milk during the early afternoon on days 2 and 3 reflected acute effects of maternal supplementation. Nevertheless, the course of the day appeared to be sufficient to negate these acute supplementation effects when the supplement was consumed in the early morning (0800–0859) and allowed the collection of samples that represented the daily median vitamin concentration during the evening. Due to the observed time between supplement consumption and concentration increases in the vitamins, any supplement consumption occurring later in the day is expected to shift the timing of a representative sample collection toward later hours in the evening. Whether or not evening consumption of supplements allows for representative milk samples in the morning has yet to be determined. Given the metabolic differences between the active and the resting hours, vitamin secretion might also be subjected to the slower metabolism. The acute response of riboflavin and vitamin B-6 also enabled the estimate of *1*) a maximum dose of these vitamins in the milk during maternal supplementation and, subsequently, *2*) a maximum amount provided to the infant. Such information could aid in kinetic studies that include the rate and amount of vitamin uptake into breast milk and subsequent availability to the infant.

Little information is available with regard to the mechanisms of vitamin secretion into breast milk (35). Water-soluble vitamins are thought to be actively secreted involving members of the solute carrier (SLC) and ATP-binding cassette (ABC) family; breast cancer–resistant protein (BCRP) is associated with riboflavin secretion, vitamin B-12 uptake might be partly dependent on a receptor-mediated system, and thiamin transporters THTR1 and THTR2 may be involved in thiamin secretion. The transport mechanisms involved with niacin and vitamin B-6 are unknown (11, 35). The regulation of fat-soluble vitamins A and E appears to occur through a specific transport system independent from lipid transfer, but the mechanisms involved have not been revealed (36, 37).

Given that only 0.05–6.17% of the detectable acute median responses to the supplemented vitamins were reflected in the milk, the uptake of the single-dose supplement into milk was very limited. Nevertheless, in the case of vitamin B-6, this limited secretion accounted for ∼30–100% of the value assumed to set the Adequate Intake for infants aged 0–6 mo. Moreover, we found that the acute effects of maternal supplementation remained significant for riboflavin and vitamin B-6 in the fasting milk samples 24 h after supplementation compared with day 1, and that the vitamins were not secreted into milk at the same rate throughout a day during maternal supplementation, resulting in a bolus dose of ≤35% of the Adequate Intake for vitamin B-6 during a single feeding, whereas the bolus dose for riboflavin during a single feeding only provided ∼11% of the Adequate Intake. These results showed that the relative increases in milk vitamin concentrations were substantial and likely to improve infant status. In addition, the reported fact that maternal supplementation caused a subsequent change in infant status of some vitamins, which reflects the amount supplied to the mother, supported the effective enrichment of these vitamins in milk.

In conclusion, diurnal variation in and timing of sample collection within a feeding are both significant but are only minor contributors to the total variance. However, the time of the recent supplement administration is crucial for deciding on the timing of breast-milk collection when establishing representative values for milk micronutrient concentrations and the effectiveness of micronutrient programs. Further evaluation of vitamin secretion into milk is needed to examine the relation between the dose and frequency of maternal supplementation and to fully understand the potential efficacy of supplements for improving the delivery of vitamins to the infant.

## Footnotes

8 Abbreviations used: ABC, ATP-binding cassette; BCRP, breast cancer–resistant protein; ICDDR,B, International Centre for Diarrhoeal Disease Research, Bangladesh; SLC, solute carrier; THTR1, thiamin transporter 1; THTR2, thiamin transporter 2; vitamin A^fat^, vitamin A adjusted for milk fat; vitamin E^fat^, vitamin E adjusted for milk fat.
